# The uphill journey of smoking cessation in chronic obstructive pulmonary disease: why a well-built vehicle matters

**DOI:** 10.3389/frhs.2025.1659295

**Published:** 2025-09-10

**Authors:** Rasmus Kragh Jakobsen, Ingeborg Farver-Vestergaard, Anders Løkke

**Affiliations:** ^1^ Department of Medicine, Lillebaelt Hospital, Vejle, Denmark; ^2^ Department of Regional Health Research, University of Southern Denmark, Odense, Denmark

**Keywords:** chronic obstructive pulmonary disease, tobacco smoking, smoking cessation, pharmacological interventions, behavioural counseling, long-lasting abstinence, mini review

## Abstract

**Introduction:**

Smoking cessation remains among the most effective interventions for improving outcomes in patients with chronic obstructive pulmonary disease (COPD). Quitting smoking slows disease progression, reduces morbidity, improves quality of life and increases life expectancy. However, a substantial proportion of patients with COPD continue to smoke, and generic cessation strategies often fall short in this population. While most cessation research targets “healthy” smokers, individuals with COPD face additional challenges – including higher nicotine dependence and psychological comorbidities – that complicate quit attempts.

**Methods:**

This mini-review summarises randomised controlled trials (RCTs) investigating smoking cessation interventions in COPD.

**Results:**

Our study reveals wide variability in the intensity, duration and components of interventions, with only a minority achieving long-term abstinence. Notably, two high-performing studies stand out for their comprehensive, long-term and individualised approaches. These findings suggest that success in smoking cessation for patients with COPD relies not only on the right intervention components but also on the construction, durability and sustained support.

**Conclusion:**

To support and sustain smoking cessation among patients with COPD, multicomponent, high-intensity and long-duration interventions tailored to individual needs appear to be required, with an emphasis on ongoing support and frequent follow-up.

## 1 Introduction

Chronic obstructive pulmonary disease (COPD) is a leading cause of morbidity and mortality worldwide, and smoking is its most significant modifiable risk factor (1–3). It is well-established that smoking cessation is among the most effective existing interventions to halt disease progression, reduce symptom burden, improve quality of life and prolong survival (2–5). Despite this, smoking prevalence remains high among patients with COPD (6). This raises a vital question: Why are existing cessation interventions not effective in this high-risk population?

Traditional smoking cessation strategies – centered on counseling, pharmacotherapy (i.e., varenicline, buproprione, cytisine) and nicotine replacement therapy (NRT) – are generally developed and tested in otherwise healthy smokers (7, 8). While some of these interventions have been extended to patients with COPD (4, 9), this population often presents with more severe nicotine dependence, psychological distress and lower self-efficacy (10, 11). These factors may render standard cessation strategies insufficient and suggest the need for targeted approaches.

To illustrate, one may liken smoking cessation interventions to vehicles helping the patients ascend a steep incline. While most vehicles have the necessary components – wheels (NRT), engine (pharmacotherapy) and steering (behavioural support) – only some are built with the structural integrity and endurance required to complete the journey. To better understand what enables longterm cessation among smokers with COPD, we conducted a mini-review of randomised controlled trials (RCTs) evaluating smoking cessation interventions in this population.

## 2 Methods

We performed a mini-review to map the characteristics and effectiveness of RCTs targeting smoking cessation in patients with COPD. Following the Joanna Briggs Institute guidelines (12), our protocol was registered with the Open Science Framework (https://osf.io/md9ab).

Using a comprehensive block search strategy combining terms for COPD, smoking cessation interventions and cessation outcomes, we searched the databases of Medline, Embase and CINAHL from their inception to November 2024, combining the following search terms:

Block 1: (chronic obstructive lung disease OR COPD OR Chronic Obstructive Pulmonary Dis* OR Chronic Obstructive Air* Dis* OR COAD OR Airflow Obstruction?, Chronic OR Chronic Air* Obstruction? OR chronic obstructive bronchopulmonary Dis* OR chronic obstructive lung Dis* OR chronic obstructive respiratory Dis* OR chronic pulmonary obstructive dis* OR lung chronic obstructive Dis* OR lung dis*, chronic obstructive OR obstructive chronic lung Dis* OR obstructive chronic pulmonary Dis* OR obstructive lung Dis*, chronic OR pulmonary Dis*, chronic obstructive OR Asthma-Chronic Obstructive Pulmonary Disease Overlap Syndrome OR acute exacerbations of chronic bronchitis OR AECB OR Bronchitis, Chronic OR Chronic Bronchitis OR Pulmonary Emphysema).

AND

Block 2: (counseling OR e-counseling OR motivational interviewing OR nicotine replacement therapy OR varenicline OR amfebutamone OR Directive Counsel?ing OR Counsel?ing, Directive OR Prescriptive Counsel?ing OR Counsel?ing, Prescriptive OR Motivational Interview* OR Interview*, Motivational OR Distance Counsel?ing OR Counsel?ing, Distance OR E-Counsel?ing OR Ecounsel?ing OR E-Therap* OR ETherap* OR Online Counsel?ing OR remote Counsel?ing OR tele Counsel?ing OR Nicotine Replacement Therap* OR Therap*, Nicotine Replacement OR vareniclin? OR Chantix OR Champix OR vareniclin? tartrat? OR tyrvaya OR buprop* OR Amfebutamon? OR zyban OR Wellbutrin OR Quomen OR Zyntabac OR aplenzin OR budep* OR buxon OR elontril OR forfivo OR odranal OR quomem OR wellbatrin OR wellbutrin).

AND

Block 3: (smoking cessation OR Cessation?, Smoking OR Smoking Cessation? OR Giving Up Smoking OR Smoking?, Giving Up OR Up Smoking, Giving OR Quit* Smoking OR Smoking, Quitting OR * Smoking OR Smoking, Stopping OR abstination, smoking OR abstinence from nicotine OR abstinence from smoking OR abstinence from tobacco OR dehabituation, smoking OR nicotine abstin* OR nicotine cessation OR nicotine withdrawal OR smoking abstinence OR smoking dehabituation OR tobacco-use cessation).

The full search protocol as well as a PRISMA flowchart can be found in the Supplementary Material. Additional studies were identified through forward citation searches and comparison with prior systematic reviews (4, 13). We included studies published in English or Nordic languages that evaluated behavioural and/or pharmacologic cessation interventions in adults with a confirmed diagnosis of COPD. References were excluded if they were duplicates, protocols, reviews, conference abstracts and session posters, studies on animals or cells and mixed interventions other than smoking cessation. Studies were also excluded if they were not placebo-controlled RCT's or if the study focus was for another chronic disease and the COPD specific data could not be separated but included studies on patients with mixed morbidities (COPD and co-morbidities).

## 3 Results

We identified 15 placebo-controlled RCTs (5, 14–27) that investigated smoking cessation in patients with COPD, involving a total of 11.432 participants (Table 1). The studies were generally similar in terms of participant demographics (age, sex, number of pack-years) and disease severity [represented all GOLD grades (2) with an overweight of grade I and II].

**Table 1:** Characteristics of the included studies.

Study		Population						Counseling		Medical		Setting			Duration total		Outcome	
Author (year)	Country	Participants (n)	Male (%)	Mean age (years)	COPD GOLD I-IV	Mean packyears	Mean FTND	Intensity	Complexity	NRT	Var., Bup., Nor.	Inpatient	Outpatient	GP	Intervention med./couns. (weeks)	Follow-up (weeks)	Smoking cessation	Absolute benefit increase (ABI) (95% CI), *p*
Pederson (1991) (14)	USA	64	69	53,4	NA	NA	NA	OO	OO			X			-/2	26	I: 33% C: 21% PP26	ns
Anthonisen (1994) (5)	USA Canada	5,887	63	48,5	I, II	40,4	NA	OOOO	OOO	X			X		26/52	260	I: 28% C: 7% CA52	21% (19%–23%) *p* < 0.0001
Tashkin (2001) (15)	USA	278	55	54	I: 85% II: 15%	52	7	OO	OO		X		X		12/12	26	I: 16% C: 9% CA26	7% (1%–13%) *p* = 0.04
Wagena (2005) (16)	Netherlands	220	49	51,2	“0”: 43,5% I: 21,1% II: 31,7% III: 3,5%	NA	6	OO	OO		X		X		12/12	26	I: 27% C: 8% CA26	19% (4%–34%) *p* = 0.02
Tonnesen (2006) (17)	Denmark	288	48	61	I: 9% II: 53% III: 30% IV: 8%	42,7	6,2	OO	OO	X			X		12/52	52	I: 14% C: 5% CA52	9% (3%–15%) *p* = 0.03
Christenhusz (2007) (18)	Netherlands	225	52	58	II, III	44	5,4	OOOO	OO		X		X		12/30	52	I: 19% C: 9% CA52	10% (1%–19%) *p* = 0.014
Sundblad (2008) (19)	Sweden	391	46	53	I: 71% II: 23% III: 6%	34,4	4,8	OOOOO	OOOOO	X		X	X		52/52	156	I: 52% C: 7% PP52	45% (37%–53%) *p* < 0.0001
Wilson (2008) (20)	N. Ireland	68	48	61	II	41,4	NA	OOO	O				X		-/5	52	I: 0% C: 0% CA52	ns
Kotz (2009) (21)	Netherlands Belgium	250	62	53,9	I: 54% II: 46%	43,2	4,5	OOO	O		X		X		7/3	52	I: 11% C: 6% CA52	ns
Tashkin (2011) (22)	USA Spain France Italy	499	62	57	I: 22,5% II 66,7% III: 10,4% IV: 0,4%	49	6,1	OOO	OOO		X		X		12/52	52	I: 19% C: 6% CA52	13% (7%–19%) *p* < 0.0001
Lou (2013) (23)	China	2,607	48	61,5	I, II, III, IV	37,7	5,1	OOOOO	OOOOO				X	X	-/104	208	I: 46% C: 4% CA52	42% (39%–45%) *p* < 0.0001
Chen (2014) (24)	China	80^a^	96	61,5	I: 11% II: 46% III: 33% IV: 10%	42	4,1	OO	OO				X		-/21	26	I: 41% C: 19% CA26	22% (3%–41%) *p* = 0.012
Lei (2020) (25)	China	94	97	61	II	40	NA	OO	OO				X		-/26	520	I: 18% C: 4% CA26	14% (2%–26%) *p* = 0.011
Le Mao (2020) (26)	France	69	61	57	NA	NA	5,2	OOO	OO		X		X		12/52	52	I: 26% C: 26% CA52	ns
Tønnesen (2022) (27)	USA +15^b^	412^a^	40	57	NA	40	6	OOO	O	X	X		X		12/24	24	I: 15% C: 5% CA24	10% (2%–18%) *p* = 0.008
TOTAL:	27	11,432							14	4	7	2	14	1				

The key information that set both top performers a part is highlighted in light yellow. n = number; X = present; O = grade (1–5), Intensity of counseling is an expression for total duration and number of sessions/contacts; Complexity of counseling is an expression for the number of counseling components (written material, group/individual sessions, educational program, different professionals involved, spouses); NA, data not available; FTND, Fagerstrom Test for Nicotine Dependence; NRT, nicotine replacement therapy; Var., Varenicline; Bup., Bupropion; Nor, Nortriptyline; GP, general practice; Community, Community Smoking Treatment Units or Health Centers; med./couns., medical (NRT and/or medicine) and counseling duration respectively; PP26, point prevalence at 26 weeks = proportion of population abstinent at time asked; CA26, continuous abstinence at 26 weeks = proportion of population being abstinent for the entire duration; CI95, 95% confidence interval; *p*, *p*-value (significance level); I, intervention group; C, control group; ns, no statistically significant difference between Intervention and Control group (*p* > 0,05);

^a^ The number of participants w. COPD in a study also including ‘healthy’ smokers;

^b^ Countries: Argentina, Australia, Brazil, Bulgaria, Canada, Chile, Denmark, Finland, Germany, Mexico, New Zealand, Russia, Slovakia, South Africa, Spain.

**Figure 1:**
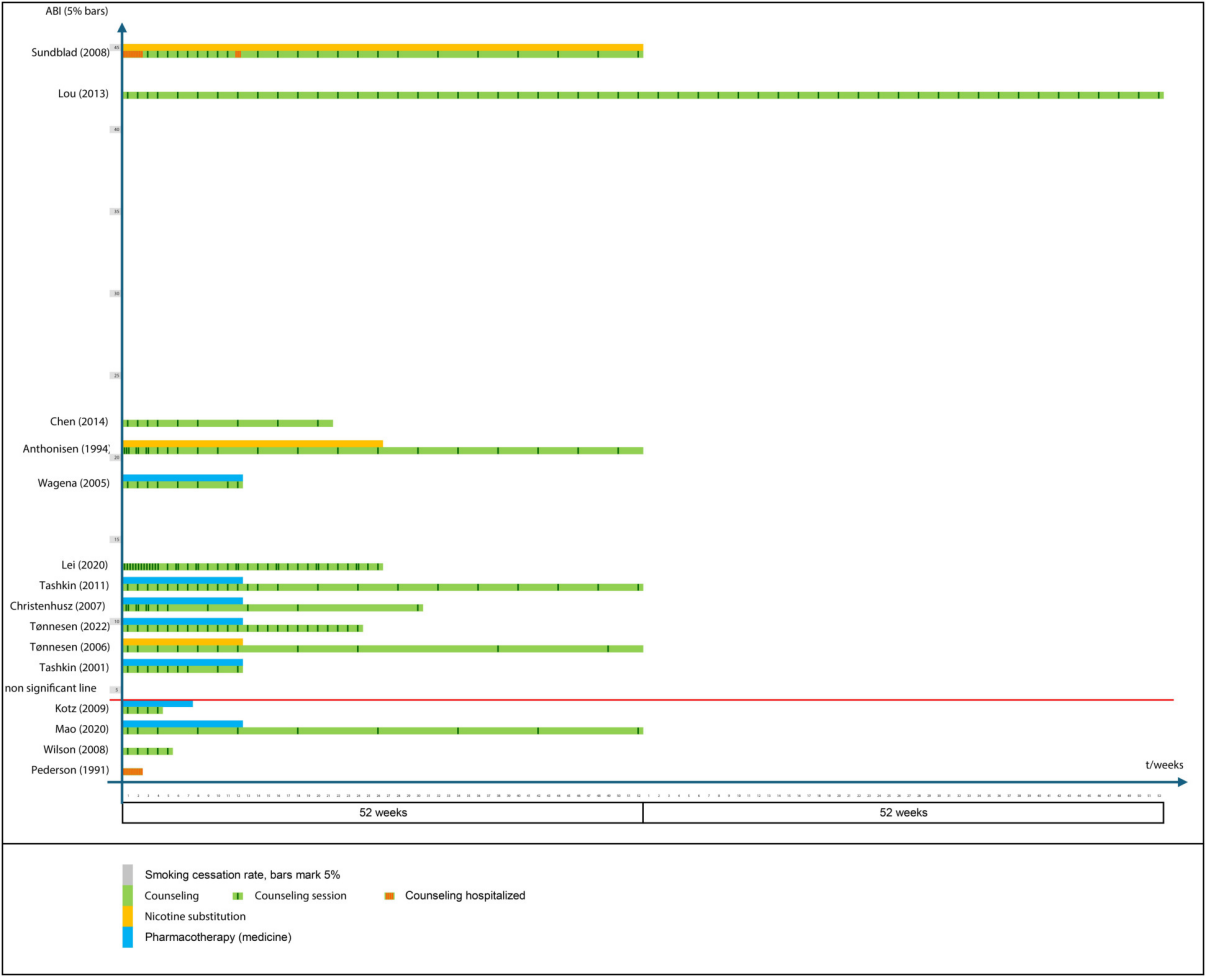
Interventions, time and smoking cessation success compared. 15 RCT's from 1991 to 2022. (1) axis is time in weeks, (2) axis is absolute benefit increases (ABI). Studies are arranged in order within 5% intervals of smoking cessation rates (grey bars). The red “non significant line” is added to mark the 4 studies where no statistically significant difference was seen between the intervention and control group (ie. ABI = 0%). Intervention types are counseling (green), nicotine replacement therapy (orange) and pharmacotherapy (blue). For studies reporting both NRT and medicines only the highest ABI is shown. To illustrate counseling intensity vertical lines marks each contact point whether individual or group sessions, phone call or text messages. Red vertical lines signifies hospitalised counseling.

All studies used counseling, six studies used pharmacotherapy in combination with counseling, three studies used NRT in combination with counseling, and one study combined all three components. Most medicine interventions lasted 12 weeks, though NRT-protocols extended up to 52 weeks. The specifics of the intervention delivery varied widely across studies (Figure 1):

• Duration ranged from 2 weeks to 2 years
• Intensity varied from brief advice (<60 min) to 11-day inpatient programs
• Number of sessions ranged from 4 to 26
• Healthcare professionals delivering the interventions included doctors, nurses, psychologists and/or trained counselors
• All studies used an outpatient setting except one study with an inpatient setting, and two studies combined the outpatient setting with either hospitalisation or visits by general practitioners. None used a community setting

Four studies followed the participants long-term (≥3 years) while the remaining studies followed participants for 12 months or less. Reported continuous abstinence (CA) rates ranged from 0% to 53%, with absolute benefit increases (ABI) of 0%–45% over control groups (Figure 1). Thirteen studies showed modest ABI (0%–22%) while two studies reported markedly higher ABIs of 42% and 45% (19, 23). These two studies also offered the most intensive, multicomponent and long-duration interventions. Hence, the study by Sundblad et al. (19) delivered an intervention that included bringing the patients (in groups of 4–10) to the hospital for smoking cessation during an 11 day stay. The stay included NRT, physical exercise and 1-hour daily meetings with a trained cessation nurse (individual counseling) and in addition a structured educational program on nicotine, health effects, dietary education, physical training, lung function testing delivered by a doctor, physiotherapist, dietitian, laboratory technician, psychologist, occupational therapist, and nurse. At home the intervention continued for 2–3 months with weekly telephone calls of 5–30 min (nurse). Then a second hospitalization for 2–4 days with spouses invited that included group discussions on how to sustain abstinence and avoid relapse. Then 10 months at home with first bi-weekly and later monthly telephone contacts. The study by Lou et al. (23) tested an intervention that included training more than 100 general practitioners in behavioural interventions for quitting to enable them to better supervise and advice the patients. The patients received individual counselling both in the general practice and during home visits by the general practitioners once per week for the first month and then at least once a month for the remainder of the intervention. Additionally, the patients were asked to participate in monthly group discussions and share the experience of quitting as well as participate in bi-monthly education by a multidisciplinary group of experts including respiratory, rehabilitation, nutrition, sports, and psychology specialists who joined the group meetings. The treatment lasted two years.

It should be noted that a Cochrane review (4) on smoking cessation for people with COPD recommends caution in the interpretation of the results of the Sundblad et al. (19) because of risk of bias due to lack of “blinding of participants and personnel”, “blinding of outcome assessment”, “incomplete outcome data” and “other bias”. However, “blinding of participants and personnel” is not possible for behavioral interventions nor for the “blinding of outcome assessment” when abstinence is self-reported. The review did find a low risk of bias due to “random sequence generation”, “allocation concealment” and “selective reporting” for Sundblad et al. and the Lou et al. (23) study was found to have an unclear risk of bias since four components of the risk of bias was unclear. The risk of selective reporting was assessed as low.

Overall, 12 of the 15 studies we found are included in the Cochrane review and almost all show either high or unclear risk of bias with the same four components as Sundblad et al. as well as low risk of bias for the same three components. This suggests an overall comparable methodological quality and a risk of bias that is at least part due to studying behavioral interventions.

## 4 Discussion

It is already well-established that a combination of behavioural and pharmacotherapeutic interventions are superior to single-component approaches in COPD (13). However, despite the consistent inclusion of counseling, pharmacotherapy and/or NRT across studies in the present review, there was substantial heterogeneity in how these components were implemented. No single combination of components appeared to consistently lead to successful, sustained outcomes, suggesting that effectiveness may depend more on how interventions are delivered than on what they contain.

### 4.1 What sets the top performers apart?

Two studies – conducted by Sundblad et al. and Lou et al. – demonstrated superior outcomes with ABIs exceeding 40% [45% (37%–53%, *p* < 0.0001) and 42% (39%–45%, *p* < 0.0001) respectively]. Though methodologically and contextually distinct, they shared a number of features:

• High intensity behavioural support
• Multidisciplinary involvement
• Long intervention duration (≥1 year)
• Frequent follow-up and patient engagement
• Broad educational and psychosocial support

Lou et al. used monthly home visits and group sessions with multidisciplinary input over two years. Sundblad et al. conducted an 11-day inpatient program followed by structured outpatient follow-up, including family involvement. While the generalisability and methodological rigor of these studies warrant further scrutiny (e.g., potential biases and use of point prevalence), their success point towards the value of long-term, individualised and intensive intervention frameworks.

None of the other 13 studies used as comprehensive or intensive interventions but within this group we may notice a similar trend: The four studies with the longest counseling duration (52 weeks) display a trend of higher outcome ABI corresponding with the level of intensity of the counseling from the highest and to the lowest (Anthonisen et al.; Tashkin et al.; Tønnesen et al.; Mao et al.) And conversely, we also notice how the three studies using the briefest counseling interventions (Pederson et al., Wilson et al. and Kotz et al.) exhibit the lowest outcome ABI.

Two studies seem to contradict the trend at a first glance (Figure 1). The study by Lei et al. displays relatively low outcome ABI compared to the very high number of counseling contacts. However, most of these contacts were text-messages which may not have as strong an effect as face-to-face or telephone contacts. The Chen et al. study display a high outcome ABI compared to the relatively short duration and low intensity, however, it should be noted that the outcome was measured after 26 weeks not 52 weeks like fx Anthonisen et al. and since the quit-rates tend to fall over time the ABI is falsely high in comparison. Similarly, the outcome ABI's reported by Wagena et al., Lei et al., Christenhusz et al., Tønnesen et al. (2022), Tashkin et al. (2001) would be expected to be lower at 52 weeks.

As pointed out in a recent scoping review of smoking cessation interventions in COPD (28), well-controlled clinical trials and rigorous, large-scale observational studies with long-term follow-up are needed to determine the optimal pharmacotherapy and the most cost-effective modalities of comprehensive smoking cessation interventions.

### 4.2 The role of structure and support

Returning to the “vehicle” metaphor, all interventions included in the present review were constructed with the necessary parts, but only a few were built to endure the challenging terrain of COPD-related smoking addiction. Hence, effective cessation in this population may require early-phase intensity to overcome withdrawal, ongoing support to prevent relapse, family and community involvement for sustained motivation, and tailored behavioural strategies responding to psychological burden and needs. Short-term interventions may capture initial abstinence but fail to support long-term cessation. The declining quit rates in studies with longer follow-up suggest that prolonged abstinence may be overestimated in brief interventions.

Intensive, long-term contact may provide psychological benefits beyond smoking cessation specifically. Due to severe addiction, most patients need several attempts to succeed with sustained smoking abstinence (29), and patients may feel “seen” and supported in this long and winding process when clinicians are personalising support strategies. Such an approach has the power to challenge common fatalistic attitudes that quitting is of no use once the disease, has developed (30) and to support the patients in converting unhelpful shame and guilt about smoking into helpful actions (i.e., attempts to quit) (31). Moreover, these interventions may signal how crucial cessation is, which can counter-act the therapeutic nihilism that is oftentimes associated with advanced COPD (32). A recent study by Zimmermann et al. (33) shows that patients with COPD, irrespective of age, sex, health literacy and burden of disease, welcome information about their illness and how to manage it – also when it comes to behavioural adaptation that require their own effort.

### 4.3 Financial considerations and reimbursement plan

Comprehensive interventions are undoubtedly more resource-intensive. However, their cost-effectiveness must be viewed in the context of the immense societal burden of COPD, including hospitalisations, disability and premature withdrawal from the workforce, which was recently pointed out as an immense problem across countries by the World Health Organisation (WHO) and the European Respiratory Society (ERS) (34). Of the two resource-intensive studies highlighted here only Sundblad et al. considers societal cost and show how their smoking intervention program, despite being extensive and expensive, would be cost-effective within a few years in the Swedish society. More specifically they calculate the cost at the time to render one smoke-free person at the 3-year follow-up to approximately 13.400 US$ and compare this to the estimated yearly societal cost of 790 US$, 4.100 US$ and 10.332 US$ for a person with mild, moderate and severe COPD respectively. In other words, halting progression makes economic sense to society not to mention the health and quality of life benefits to the patients.

Notably, inpatient rehabilitation is routinely used in other addiction disorders (34), and smokers with COPD, facing a comparably lethal and costly addiction, are rarely offered similar treatment intensity. Addressing this disparity will require structural changes, including cross-sector reimbursement models that reduce fragmentation and support patients along their cessation journey.

Initiatives like the London Tobacco Alliance's toolkit for pharmacotherapy commissioning pathways (35) as well as professional training resources developed by the National Centre for Smoking Cesssation and Training (NCSCT) in the UK (36), illustrate promising approaches to integration of care at the system-level.

### 4.4 Limitations

Several limitations should be acknowledged. First, the included studies varied significantly in methodological quality, definitions of abstinence (e.g., point prevalence vs. continuous), and length of follow-up, which complicates direct comparisons and generalizability. Second, publication bias cannot be ruled out, as interventions are more likely to be reported if successful. Third, patient characteristics such as socioeconomic status, comorbidities and health literacy were not systematically accounted for, yet these may significantly influence cessation outcomes.

## 5 Conclusion

It seems like we already know the important components of smoking cessation: counseling, NRT and pharmacotherapy. But the question is why don't they work better for patients with COPD? Existing studies varies widely in their specific delivery features, and evidence from two standout studies suggests that higher long-term quit rates are achievable through a combination of longer duration, higher intensity and individualised support. COPD-specific cessation strategies must move beyond standard protocols and towards tailored interventions that reflect the unique challenges of this population.

There is cause for cautious optimism. With strategic refinement and investment, cessation programs can be optimised to help more patients with COPD overcome addiction, improve their quality of life, and reduce the broader public health burden. Future research should systematically explore the relative contributions of intervention duration, complexity and support structure in achieving lasting abstinence in this particular patient group.
