# Supplementary material for: The uphill journey of smoking cessation in chronic obstructive pulmonary disease: why a well-built vehicle matters
Source: Front Health Serv. 2025 Sep 10;5:1659295. doi: 10.3389/frhs.2025.1659295 (PMC12457438; doi:10.3389/frhs.2025.1659295)
Supplement: Supplementary file 2 [file Datasheet2.pdf]

PRISMA flowchart. **The uphill journey of smoking cessation in chronic obstructive pulmonary disease: Why a well-built vehicle matters.**

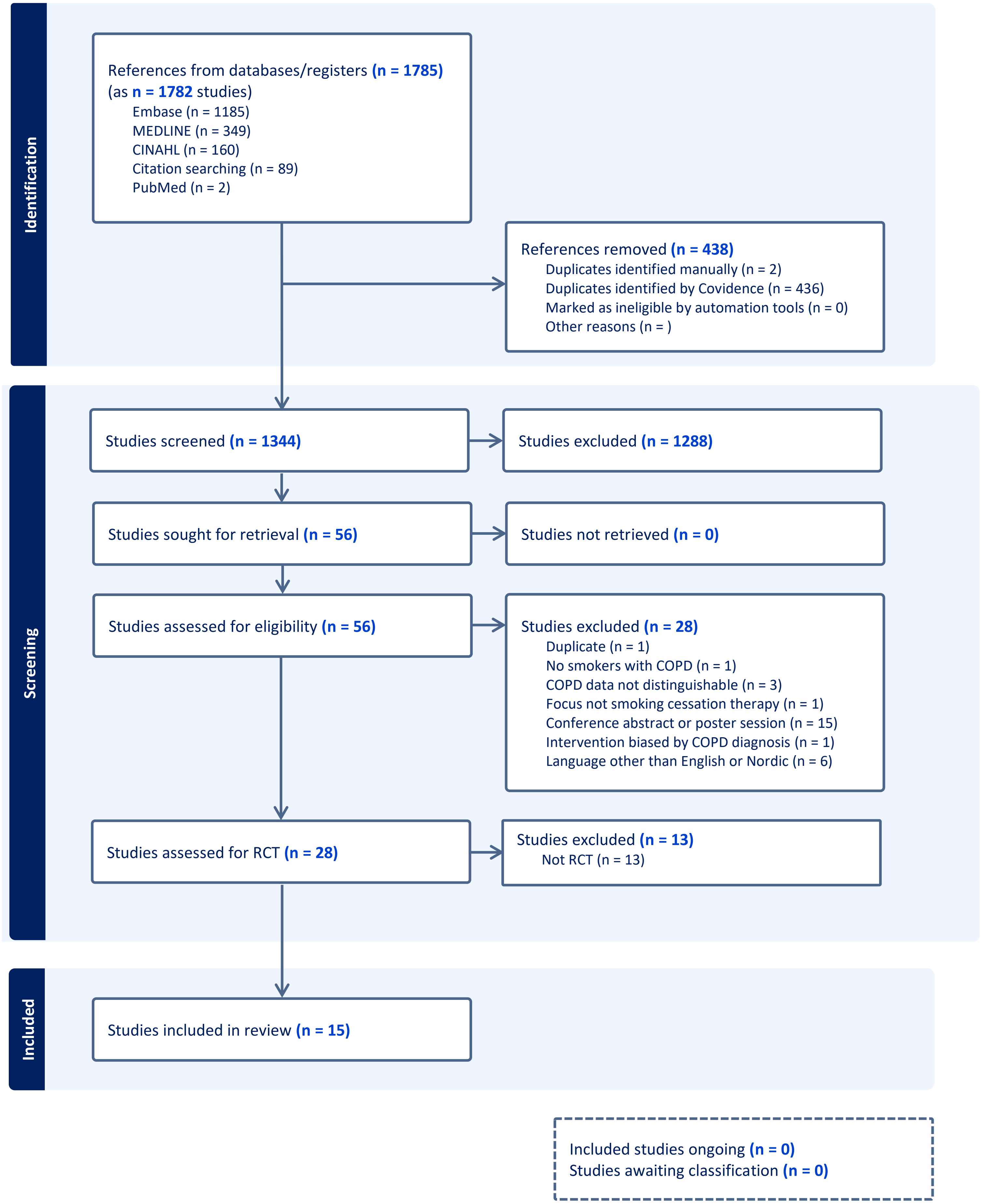

13th December 2024

covidence
